# Predictors of 30-Day Mortality and Morbidity Following Craniotomy for Traumatic Brain Injury: An ACS NSQIP Database Analysis

**DOI:** 10.1089/neur.2024.0039

**Published:** 2024-07-16

**Authors:** Jawad Turfa, Ali Hijazi, Yasser Fadlallah, Melhem El-Harati, Hani Dimassi, Marwan El Najjar

**Affiliations:** ^1^ Faculty of Medicine, American University of Beirut, Beirut, Lebanon.; ^2^ School of Pharmacy, Lebanese American University, Byblos, Lebanon.; ^3^ Division of Neurosurgery, Department of Surgery, American University of Beirut Medical Center, Beirut, Lebanon.

**Keywords:** ACS NSQIP, craniotomy, morbidity, mortality, traumatic brain injury

## Abstract

Traumatic brain injury (TBI) is the leading cause of death among trauma patients. Identifying preoperative factors that predict postoperative outcomes in such patients can guide surgical decision-making. The aim of this study was to develop a predictive model using preoperative variables that predicts 30-day mortality and morbidity in patients undergoing neurosurgery following TBI. The American College of Surgeons National Surgical Quality Improvement Program (ACS NSQIP) database was queried between 2005 and 2017 for patients aged 18 years or older who underwent TBI-specific surgery. The primary outcome was 30-day mortality, and the secondary outcome was a composite morbidity score. Significant variables on univariate analysis with Chi-squared test were used to compute multivariable logistic regression models for both outcomes, and Hosmer–Lemeshow test was used. A total of 1634 patients met the inclusion criteria. Most patients were elderly aged >60 years (74.48%), male (63.59%), of White race (73.62%), and non-Hispanic ethnicity (82.44%). The overall 30-day mortality rate was 20.3%. Using multivariate logistic regression, 11 preoperative variables were significantly associated with 30-day mortality, including (aOR, 95% CI) age 70–79 years (3.38, 2.03–5.62) and age >80 years (7.70, 4.74–12.51), ventilator dependency (6.04, 4.21–8.67), receiving dialysis (4.97, 2.43–10.18), disseminated cancer (4.42, 1.50–13.0), and coma >24 hours (3.30, 1.40–7.80), among others. Similarly, 12 preoperative variables were found to be significantly associated with 30-day morbidity, including acute renal failure (7.10, 1.91–26.32), return to OR (3.82, 2.77–5.27), sepsis (3.27, 1.11–9.66), prior operation within 30 days (2.55, 1.06–4.95), and insulin-dependent diabetes (1.60, 1.06–2.40), among others. After constructing receiver operating characteristic curve, the model for mortality had an area under the curve (AUC) of 0.843, whereas composite morbidity had an AUC of 0.716. This model can aid in clinical decision-making for triaging patients based on prognosis in cases of mass casualty events.

## Introduction

Traumatic brain injury (TBI) is defined as an alteration in brain function, or other evidence of brain pathology, caused by an external force.^1^ TBI is the leading cause of injury related mortality and morbidity, accounting for 30% of all deaths related to injuries.^2^ Causes include forceful bumps, blows, head or body jolts, or objects that pierce the skull and penetrate the brain tissue.^3^ The resulting head injuries are classified according to their mechanism into the following two types: penetrating or open TBI and nonpenetrating TBI, also known as closed head injury or blunt TBI.^3^ With regards to severity, TBI is classified into mild, moderate, and severe based on the Glasgow Coma Score (GCS), which has been implemented as an objective tool to describe the extent of impaired consciousness in acute medical and/or trauma patients.^4^ Complications of head trauma such as skull fractures, intracranial bleeds, and increased intracranial pressure (ICP) often necessitate surgical intervention that can be lifesaving and/or used to limit the potential associated morbid events that follow injury.

Several preoperative factors have been used to predict mortality in patients undergoing various surgical procedures. For instance, the American Society of Anesthesiologists physical status classification system is commonly used by clinicians to predict postoperative outcomes using perioperative variables.^5^ Other examples of such systems include the Physiological and Operative Severity Score for the enUmeration of Mortality and Morbidity (POSSUM) scale, which predicts risk-adjusted mortality in a variety of surgical procedures, and the Acute Physiology and Chronic Health Evaluation II (APACHEII) severity-of-disease score, which focuses on preoperative laboratory values while taking into account patient age and chronic health evaluation within 24 h of admission to the ICU unit.^6,7^

In the field of neurosurgery, the NSQIP surgical risk calculator remains a valuable tool for evaluating postoperative risk.^8^ However, valid predictive models specific to neurosurgical procedures are still lacking. The case is particularly relevant for patients undergoing emergent surgeries for acute TBI such as emergent evacuation of a subdural hematoma or craniotomy to alleviate elevated ICP. As the morbidity and mortality rates in this patient population remain high, it is essential to identify factors that predict postoperative outcomes to aid in prognostic and surgical judgment, which would prove useful in under-resourced institutions or mass-casualty events where patient triage is of the essence. In this study, we aimed to develop a model based on the American College of Surgeons National Surgical Quality Improvement Program (ACS NSQIP) database that can efficiently predict 30-day mortality and morbidity in patients undergoing neurosurgery for acute TBI.

## Materials and Methods

### Data source

The ACS NSQIP neurosurgical procedure database was used for this study. The ACS NSQIP collects data on over 150 variables, including preoperative risk factors, intraoperative variables, and 30-day postoperative mortality and morbidity outcomes for patients undergoing major surgical procedures in both the inpatient and outpatient setting.^9^ A site’s trained and certified Surgical Clinical Reviewer captures these data using a variety of methods, including medical chart abstraction.^9^ In this study, we used the neurosurgical subset of the database, which included a total of 332,971 cases.

### Study population

**FIG. 1.**
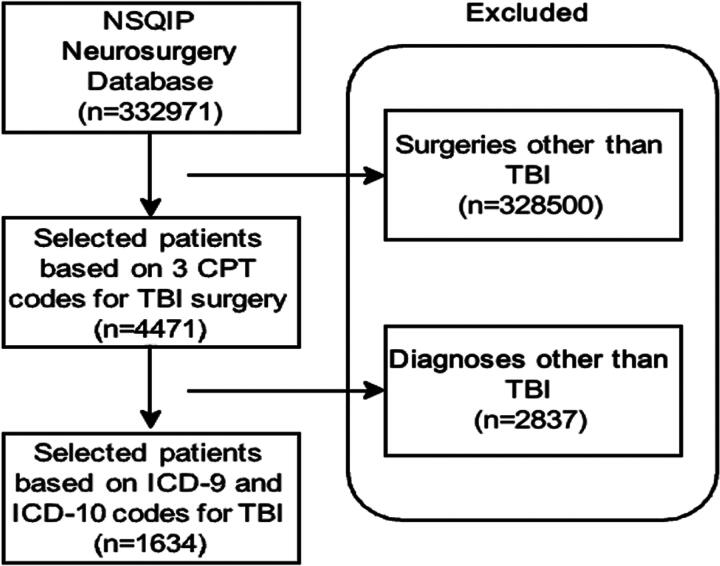
Flow diagram depicting study patient selection. NSQIP: National Surgical Quality Improvement Program, TBI: Traumatic Brain Injury, CPT: Current Procedural Terminology, ICD-9: International Classification of Diseases Ninth Revision, ICD-10: International Classification of Diseases Tenth Revision.

Patients 18 years or older who underwent TBI-related surgeries were selected from the database. The CPT (Current Procedural Terminology) codes used were 61312, 61322, and 61345 corresponding to supratentorial hematoma evacuation (extradural and subdural), decompressive craniectomy to treat raised intracranial pressure (ICP), and decompressive craniectomy of the posterior fossa, respectively. Additional filters using TBI-related diagnoses based on ICD-9 (International Classification of Diseases Ninth Revision) and ICD-10 (International Classification of Diseases Tenth Revision) were used. A total of 1,634 patients met the inclusion criteria and were included in the final analysis (Fig. 1).

### Data management and analysis

Continuous variables, including age, hematocrit, platelet count, white blood cell (WBC) count, international normalised ratio (INR), Creatinine, BMI, and length of stay, were categorized based on laboratory cutoffs or clinically meaningful subgroups. The primary outcome was 30-day mortality, whereas the secondary outcome was a composite measure of morbidity. The composite morbidity score included the following four categories of postoperative comorbidities: venous thromboembolism (VTE) composed of two variables (“occurrences pulmonary embolism” and “DVT/thrombo”), infections composed of six variables (“Occurrence of Pneumonia”, “Superficial Site Infection”, “Deep Incisional SSI”, “Organ Space SSI”, “Sepsis”, and “Urinary tract infection”), cerebrovascular accident (CVA) composed of two variables (“Myocardial infraction” and “CVA/Stroke”), and length of stay. The scoring was as follows: 1 point for any VTE, CVA, Infection, or length of stay >14 days. A final morbidity score of 0 corresponded to “No”, whereas a score of 1 or higher corresponded to “Yes.” Finally, composite morbidity was categorized into 2 categories based on the score.

Univariate analysis using chi-square test to identify covariates significantly associated with both mortality and morbidity, with a cutoff of α < 0.05 for significance, was used. Forward and backward logistic regression models with a cutoff of *p* < 0.1 were computed to identify significant predictors of mortality and morbidity. Hosmer–Lemeshow test of goodness of fit and receiver operating characteristic (ROC) curves were used to assess the predictive capacity of the models. All statistical analyses were conducted using SPSS version 29.0.

## Results

### Baseline demographics

The neurosurgical database included 332,971 patients between 2005 and 2017. A total of 1,634 patients met the inclusion criteria based on TBI diagnoses and surgical procedures and were included in the study. Figure 1 summarizes the stepwise selection of cases. The majority of patients were elderly, aged >60 years (74.48%), with the >80-year subgroup having the highest sample proportion (30.33%). The cohort was predominantly male (63.59%), of White race (73.62%), and non-Hispanic ethnicity (82.44%). Table 1 summarizes the baseline demographics of the patient cohort.

**Table 1.** Baseline Demographics and Predictors of Mortality and Morbidity with Unadjusted Odds Ratios and Significance Levels

			Mortality				Morbidity			
Variable	Categories	Count	Dead	Not dead	OR	*p* value	Yes	No	OR	*p* value
**Age**	<60	417	9.6%	90.4%	—	**<0.001**	35.5%	64.5%	—	0.928
	60–69	298	15.1%	84.9%			33.6%	66.4%		
	70–79	425	21.4%	78.6%			33.9%	66.1%		
	≥80	494	31.6%	68.4%			33.6%	66.4%		
Sex	Male	1039	19.6%	80.4%	1.12	0.364	33.0%	67.0%	1.15	0.2
	Female	595	21.5%	78.5%			36.1%	63.9%		
Body Mass Index (BMI)	Underweight (<18.5)	73	19.2%	80.8%	—	0.337	32.9%	67.1%	—	0.31
	Normal (18.5–24.9)	571	21.4%	78.6%			35.4%	64.6%		
	Overweight (25–29.9)	531	18.6%	81.4%			30.7%	69.3%		
	Obese Class 1 (30–34.9)	209	19.6%	80.4%			35.4%	64.6%		
	Obese Class 2 (35–39.9)	83	16.9%	83.1%			42.2%	57.8%		
	Obese Class 3 (>40)	33	6.1%	93.9%			30.3%	69.7%		
**Race**	White	1203	22.1%	77.9%	—	**0.018**	33.0%	67.0%	—	0.23
	Black	161	13.7%	86.3%			40.4%	59.6%		
	Others	101	18.8%	81.2%			32.7%	67.3%		
	Unknown	169	14.8%	85.2%			37.3%	62.7%		
**Diabetes**	No	1303	18.3%	81.7%	**1.78**	**<0.001**	33.3%	66.7%	1.20	0.155
	Yes	331	28.4%	71.6%			37.5%	62.5%		
**Insulin-Dependent Diabetes**	No	1516	19.8%	80.2%	1.51	0.057	33.2%	66.8%	**1.76**	**0.003**
	Yes	118	27.1%	72.9%			46.6%	53.4%		
**Hypertension Requiring Medication**	No	629	15.1%	84.9%	**1.74**	**<0.001**	28.5%	71.5%	**1.52**	**<0.001**
	Yes	1005	23.6%	76.4%			37.7%	62.3%		
**Current Smoker within One Year**	No	1360	22.3%	77.7%	**0.41**	**<0.001**	34.4%	65.6%	0.93	0.618
	Yes	274	10.6%	89.4%			32.8%	67.2%		
**Currently on Dialysis (Preop)**	No	1593	19.6%	80.4%	**3.53**	**<0.001**	33.6%	66.4%	**2.28**	**0.008**
	Yes	41	46.3%	53.7%			53.7%	46.3%		
**Bleeding Disorders**	No	1226	14.5%	85.5%	**3.57**	**<0.001**	30.8%	69.2%	**1.77**	**<0.001**
	Yes	408	37.7%	62.3%			44.1%	55.9%		
**Disseminated Cancer**	No	1608	19.8%	80.2%	**4.73**	**<0.001**	—	—	—	—
	Yes	26	53.8%	46.2%			—	—		
**Coma >24 H**	No	1617	19.6%	80.4%	**30.76**	**<0.001**	34.0%	66.0%	2.19	0.1
	Yes	17	88.2%	11.8%			52.9%	47.1%		
**Ventilator Dependent**	No	1283	12.6%	87.4%	**6.50**	**<0.001**	30.6%	69.4%	**2.01**	**<0.001**
	Yes	351	48.4%	51.6%			47.0%	53.0%		
**Prior Operation within 30 days**	No	1615	20.4%	79.6%	0.73	0.622	33.8%	66.2%	**3.34**	**0.007**
	Yes	19	15.8%	84.2%			63.2%	36.8%		
**Emergency Case**	No	535	9.0%	91.0%	**3.54**	**<0.001**	24.5%	75.5%	**1.96**	**<0.001**
	Yes	1099	25.8%	74.2%			38.9%	61.1%		
**>10% Loss of Body Weight in Last 6 months**	No	1604	—	—	—	—	33.7%	66.3%	**2.57**	0.009
	Yes	30	—	—			56.7%	43.3%		
**Functional Health Status Prior to Surgery**	Independent	1349	19.1%	80.9%	—	**<0.001**	32.9%	67.1%	—	**0.003**
	Partially Dependent	189	20.1%	79.9%			34.9%	65.1%		
	Totally Dependent	96	38.5%	61.5%			50.0%	50.0%		
**Systemic Sepsis**	No	1321	17.9%	82.1%	—	**<0.001**	30.7%	69.3%	—	**<0.001**
	SIRS	288	30.6%	69.4%			47.6%	52.4%		
	Sepsis	19	21.1%	78.9%			68.4%	31.6%		
	Septic Shock	6	50.0%	50.0%			50.0%	50.0%		
**Hematocrit**	≤25	46	50.0%	50.0%	—	**<0.001**	47.8%	52.2%	—	**<0.001**
	25–40	1026	22.0%	78.0%			37.3%	62.7%		
	>40	530	14.3%	85.7%			26.6%	73.4%		
**WBC Count**	≤10	950	14.6%	85.4%	**2.29**	**<0.001**	28.4%	71.6%	**1.87**	**<0.001**
	>10	643	28.1%	71.9%			42.6%	57.4%		
**Platelet Count**	≤150	289	31.5%	68.5%	**0.47**	**<0.001**	43.9%	56.1%	**0.60**	**<0.001**
	>150	1307	17.6%	82.4%			31.9%	68.1%		
**INR**	≤1.2	1231	16.7%	83.3%	**2.93**	**<0.001**	33.5%	66.5%	1.24	0.115
	>1.2	275	37.1%	62.9%			38.5%	61.5%		
**Creatinine**	≤1.2	1311	17.2%	82.8%	**2.57**	**<0.001**	33.6%	66.4%	1.14	0.354
	>1.2	282	34.8%	65.2%			36.5%	63.5%		
**CVA/Stroke**	No	1573	19.8%	80.2%	**1.97**	**0.014**	34.1%	65.9%	1.01	0.963
	Yes	61	32.8%	67.2%			34.4%	65.6%		
Hispanic	No	1347	21.5%	78.5%	0.66	0.074	35%	65.%	0.75	0.123
	Yes	156	15.4%	84.6%			28.8%	71.2%		
2 drinks/day in 2 weeks before admission	No	1595	—	—	—	—	34.1%	65.9%	1.08	0.816
	Yes	39	—	—			35.9%	64.1%		
History of severe COPD	No	1529	20.1%	79.9%	1.18	0.504	33.9%	66.1%	1.20	0.378
	Yes	105	22.9%	77.1%			38.1%	61.9%		
**History of CHF**	No	1594	19.9%	80.1%	**2.16**	**0.019**	33.9%	66.1%	1.60	0.143
	Yes	40	35.0%	65.0%			45.0%	55.0%		
Previous PCI	No	1594	20.0%	80.0%	1.92	0.053	34.1%	65.9%	1.16	0.651
	Yes	40	32.5%	67.5%			37.5%	62.5%		
Previous Cardiac Surgery	No	1591	20.1%	79.9%	1.54	0.21	33.9%	66.1%	1.55	0.16
	Yes	43	27.9%	72.1%			44.2%	55.8%		
**Acute Renal Failure**	No	1619	20.3%	79.7%	1.43	0.539	33.7%	66.3%	**7.86**	**<0.001**
	Yes	15	26.7%	73.3%			80.0%	20.0%		
**Impaired Sensorium**	No	1502	19.2%	80.8%	**2.03**	**<0.001**	33.8%	66.2%	1.24	0.257
	Yes	132	32.6%	67.4%			38.6%	61.4%		
**Open Wound/Wound infection**	No	1560	19.9%	80.1%	**1.71**	**0.039**	33.5%	66.5%	**1.88**	**0.007**
	Yes	74	29.7%	70.3%			48.6%	51.4%		
Steroid use for Chronic Conditions	No	1588	20.1%	79.9%	1.57	0.174	34.3%	65.7%	0.84	0.59
	Yes	46	28.3%	71.7%			30.4%	69.6%		
**Transfusion >4 Packed RBCs**	No	1596	20.0%	80.0%	**2.08**	**0.031**	34.1%	65.9%	1.13	0.723
	Yes	38	34.2%	65.8%			36.8%	63.2%		
**Return to OR**	No	1430	21.0%	79.0%	0.70	0.079	30.3%	69.7%	**3.64**	**<0.001**
	Yes	204	15.7%	84.3%			61.3%	38.0%		
Inpatient	No	9	11.1%	88.9%	2.05	0.491	33.3%	66.7%	1.04	0.959
	Yes	1625	20.4%	79.6%			34.2%	65.8%		
Dyspnea	No	1569	—	—	—	—	33.6%	66.4%	—	0.06
	At rest	24	—	—			50.0%	50.0%		
	Moderate exertion	41	—	—			46.3%	53.7%		
Wound Classification	Clean or none	1587	—	—	—	—	33.8%	66.2%	—	0.192
	Clean/Contaminated	37	—	—			45.9%	54.1%		
	Contaminated	9	—	—			44.4%	55.6%		
	Dirty/Infected	1	—	—			100.0%	0.0%		

No odds ratios were calculated for subcategories for variables with >2 categories. No univariate analysis was done against an outcome for variables having <10 cases associated with that outcome. Names of variables significantly associated with any outcome, associated Odds rations, and *p* values are in bold.

### Primary outcome (30-day mortality)

The following 23 preoperative variables were found to be significantly associated with 30-day mortality after neurosurgery for TBI on univariate analysis: age (*p* < 0.001), race (*p* = 0.018), diabetes (OR 1.78, *p* < 0.001), hypertension (OR 1.74, *p* < 0.001), current smoking within last year (OR 0.41, *p* < 0.001), currently on dialysis (OR 3.53, *p* < 0.001), bleeding disorders (OR 3.57, *p* < 0.001), disseminated cancer (OR 4.73, *p* < 0.001), coma > 24 h (OR 30.76, *p* < 0.001), ventilator dependent (OR 6.50, *p* < 0.001), emergency case (OR 3.54, *p* < 0.001), functional health status prior to surgery (*p* < 0.001), systemic sepsis (*p* < 0.001), hematocrit (*p* < 0.001), WBC count (OR 2.29, *p* < 0.001), platelet count (OR 0.47, *p* < 0.001), INR (OR 2.93, *p* < 0.001), Creatinine (OR 2.57, *p* < 0.001), CVA/stroke (OR 1.97, *p* = 0.014), history of congestive heart failure (CHF) (OR 2.16, *p* = 0.019), impaired sensorium (OR 2.03, *p* < 0.001), open wound/wound infection (OR 1.71, *p* = 0.039), and transfusion of >4 packed RBCs (OR 2.08, *p* = 0.031). Table 1 summarizes the results of the univariate analysis across the baseline and preoperative variables for 30-day mortality.

Variables that were significant on univariate analysis (*p* < 0.05) were plugged into a forward stepwise logistic regression model with a cutoff of <0.1. The model was significant (*p* < 0.001), and the Hosmer–Lemeshow goodness of fit test showed that the model fits the data well (*p* = 0.659). The model identified 11 significant predictors of 30-day mortality as follows: Age was a significant predictor of 30-day mortality (*p* < 0.001). Compared to those <60 years of age, patients in the 60–69, 70–79, and >80-year age groups were 1.66 (95% CI, 0.95–2.88, *p* = 0.074), 3.38 (95% CI, 2.03–5.62, *p* < 0.001), and 7.70 (95% CI, 4.74–12.51, *p* < 0.001) times more likely to die, respectively. Diabetic patients were 1.51 times more likely to die compared with nondiabetics (95% CI, 1.05–2.16, *p* = 0.026). Patients dependent on ventilators were 6.04 times more likely to die compared with those not on ventilators (95% CI, 4.21–8.67, *p* < 0.001). Patients receiving dialysis preop were 4.97 times more likely to die compared with those not on dialysis (95% CI, 2.43–10.18, *p* < 0.001). Patients in a coma >24 h were 3.30 times more likely to die compared with those who weren’t (95% CI, 1.40–7.80, *p* = 0.006).

**FIG. 2.**
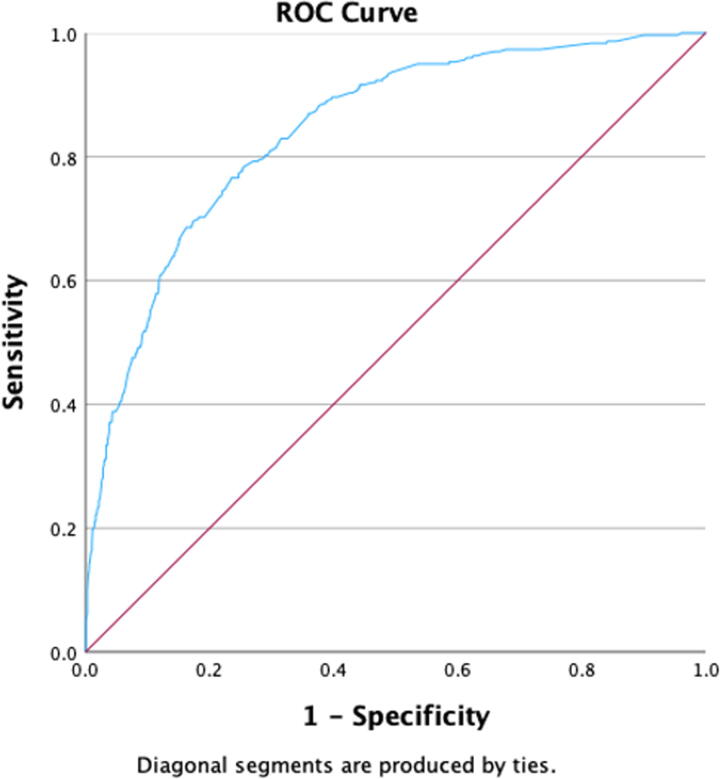
Receiver Operating Characteristic (ROC) curve of Probabilities for the Preoperative Variables and Mortality at 30 Days.

Patients with disseminated cancer were 4.42 times more likely to die compared with those who didn’t (95% CI, 1.50–13.00, *p* = 0.007). Patients with a bleeding disorder were 1.72 times more likely to die than those who didn’t (95% CI, 1.21–2.43, *p* = 0.002). Emergency cases were 2.05 times more likely to end in death compared with nonemergent cases (95% CI, 1.38–3.05, *p* < 0.001). Patients with leukocytosis (WBC > 10,000) were 1.72 times more likely to die compared with those with normal WBC count (<10,000) (95% CI, 1.24–2.38, *p* = 0.001). Patients with an elevated INR (>1.2) were 1.53 times more likely to die compared with those with normal INR (<1.2) (95% CI, 1.05–2.22, *p* = 0.027). Finally, patients with a normal platelet count (>150,000) were 0.49 times less likely to die compared with those with thrombocytopenia (platelet count < 150,000) (95% CI, 0.34–0.70, *p* < 0.001). Table 2 summarizes the results of the logistic regression model for predictors of 30-day mortality. Using the predicted probability of our model, an ROC (receiver operating characteristic) curve was constructed with an area under the curve of 0.843 (95% CI, 0.819–0.867, *p* < 0.001) and standard error = 0.012, which reflect strong discriminatory power of the model developed. The curve is shown in Figure 2.

**Table 2.** Multivariate Logistic Regression Model for Predictors of 30-Day Mortality After Undergoing Neurosurgical Procedures Following Acute Traumatic Brain Injury

Variable	Categories	AOR	95% CI	*p* value
**Diabetes**	No	Ref	—	—
	Yes	**1.51**	1.05–2.16	**0.026**
**Ventilator Dependent**	No	Ref	—	—
	Yes	**6.04**	4.21–8.67	**<.001**
**Currently on Dialysis**	No	Ref	—	—
	Yes	**4.97**	2.43–10.18	**<.001**
**Coma > 24 H**	No	Ref	—	—
	Yes	**3.30**	1.40–7.80	**0.006**
**Disseminated Cancer**	No	Ref	—	—
	Yes	**4.42**	1.5–13.00	**0.007**
**Bleeding Disorders**	No	Ref	—	—
	Yes	**1.72**	1.21–2.43	**0.002**
**Emergency Case**	No	Ref	—	—
	Yes	**2.05**	1.38–3.05	**<.001**
**Age**				**<.001**
	<60 years	Ref	—	—
	60–69 years	1.66	0.95–2.88	0.074
	70–79 years	3.38	2.03–5.62	**<.001**
	≥80 years	7.7	4.74–12.51	**<.001**
**WBC Count**	<10,000	Ref	—	—
	>10,000	**1.72**	1.24–2.38	**0.001**
**INR**	<1.2	Ref	—	—
	>1.2	**1.53**	1.05–2.22	**0.027**
**Platelet Count**	<150,000	Ref	—	—
	>150,000	**0.49**	0.34–0.70	**<.001**

Significant variable names, associated aOR, and *p* values are in bold.

### Secondary outcome (composite morbidity)

The following 16 preoperative variables were found to be significantly associated with composite morbidity after neurosurgery for TBI on univariate analysis: insulin-dependent diabetes (OR 1.76, *p* = 0.003), hypertension requiring medication (OR 1.52, *p* < 0.001), currently on dialysis (OR 2.28, *p* = 0.008), bleeding disorders (OR 1.77, *p* < 0.001), ventilator dependent (OR 2.01, *p* < 0.001), prior operation within 30 days (OR 3.34, *p* = 0.007), emergency case (OR 1.96, *p* < 0.001), >10% loss of body weight in last 6 months (OR 2.57, *p* = 0.009), functional health status before surgery (*p* = 0.003), systemic sepsis (*p* < 0.001), hematocrit (*p* < 0.001), WBC count (OR 1.87, *p* < 0.001), platelet count (OR 0.60, *p* < 0.001), acute renal failure (OR 7.86, *p* < 0.001), open wound/wound infection (OR 1.88, *p* = 0.007), and return to OR (OR 3.64, *p* < 0.001). Table 1 summarizes the results of the univariate analysis across the baseline and preoperative variables for composite morbidity.

Variables that were significant on univariate analysis (*p* < 0.05) were plugged into a backward logistic regression model with a cutoff of <0.1. The model was significant (*p* < 0.001), and the Hosmer–Lemeshow goodness of fit test showed that the model fit the data well (*p* = 0.867). The model identified 11 significant predictors of composite morbidity as follows: Patients who had insulin-dependent diabetes were 1.60 times more likely to develop a postop morbidity compared with those who didn’t (95% CI, 1.06–2.40, *p* = 0.025). Patients who had hypertension requiring medication were 1.35 times more likely to develop a post-op morbidity compared with normotensive patients (95% CI, 1.07–1.71, *p* = 0.013). Patients who were on a ventilator were 1.38 times more likely to develop a post-op morbidity compared with those not on a ventilator (95% CI, 1.04–1.82, *p* = 0.025). Emergency cases were 1.45 times more likely to produce a post-op morbidity compared with nonemergent cases (95% CI, 1.11–1.88, *p* = 0.025). Patients who had >10% body weight loss in last 6 months were 2.29 times more likely to develop a post-op morbidity compared with those who didn’t (95% CI, 1.06–4.95, *p* = 0.035).

**FIG. 3.**
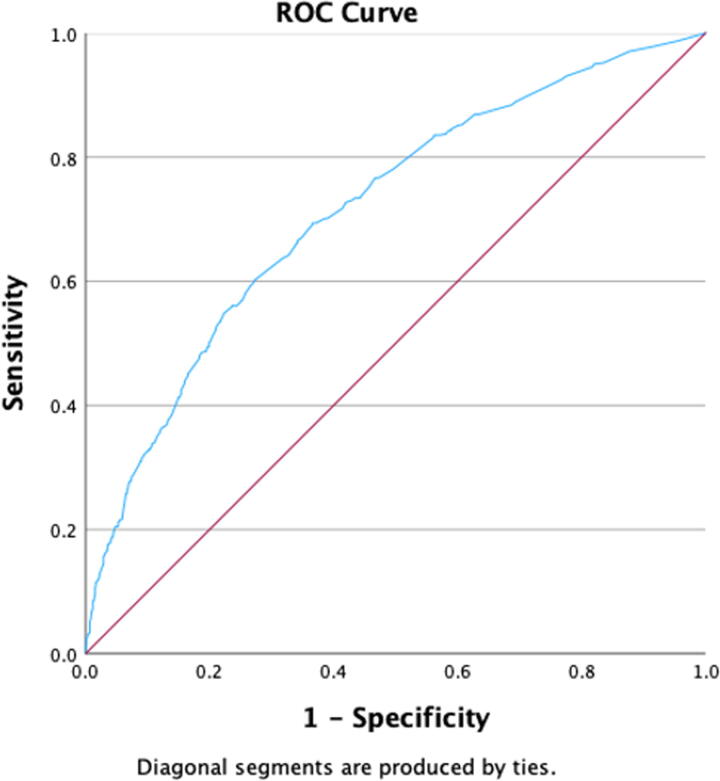
Receiver Operating Characteristic (ROC) curve of Probabilities for the Preoperative Variables and Composite Morbidity.

Patients who had SIRS were 1.59 (95% CI, 1.19–2.12, *p* = 0.002) times and those with sepsis were 3.27 (95% CI, 1.11–9.66, *p* = 0.032) times more likely to develop a post-op morbidity compared with those who didn’t, respectively. Patients with leukocytosis (WBC > 10,000) were 1.51 times more likely to develop a post-op morbidity compared with those who had normal WBC count (<10,000) (95% CI, 1.18–1.93, *p* < 0.001). Patients who had acute renal failure were 7.10 times more likely to develop a post-op morbidity compared with those who didn’t (95% CI, 1.91–26.32, *p* = 0.003). Patients with a normal platelet count (>150,000) were 0.61 times less likely to develop a post-op morbidity compared with those who had thrombocytopenia (<150,000) (95% CI, 0.46–0.81, *p* < 0.001). Finally, patients who returned to the OR were 3.82 times more likely to develop a post-op morbidity compared with those who didn’t (95% CI, 2.77–5.27, *p* < 0.001). Table 3 summarizes the results of the logistic regression model for predictors of postoperative morbidity. Using the predicted probability of our model, an ROC curve was constructed with an area under the curve of 0.716 (95% CI, 0.689–0.742, *p* < 0.001) and standard error = 0.014, which reflect moderate discriminatory power of the model developed. The curve is shown in Figure 3.

**Table 3.** Multivariate Logistic Regression Model for Predictors of Composite Morbidity After Undergoing Neurosurgical Procedures Following Acute Traumatic Brain Injury

Variable	Categories	AOR	95% CI	*p* value
**Insulin-Dependent DM**	No	Ref	—	—
	Yes	**1.60**	1.06—2.4	**0.025**
**HTN Requiring Medication**	No	Ref	—	—
	Yes	**1.35**	1.07—1.71	**0.013**
**Ventilator Dependent**	No	Ref	—	—
	Yes	**1.38**	1.04—1.82	**0.025**
Prior Operation within 30 Days	No	Ref	—	—
	Yes	2.55	0.85—7.64	0.093
**Emergency Case**	No	Ref	—	—
	Yes	**1.45**	1.11—1.88	**0.006**
**>10% Loss of Body Weight in 6 Months**	No	Ref	-	—
	Yes	**2.29**	1.06—4.95	**0.035**
Hematocrit				**0.005**
	<25	Ref	—	—
	25—40	0.78	0.41—1.50	0.46
	>40	0.53	0.27—1.04	0.065
**Systemic Sepsis**				**0.003**
	No	Ref	—	—
	**SIRS**	**1.59**	1.19—2.12	**0.002**
	**Sepsis**	**3.27**	1.11—9.66	**0.032**
	Septic Shock	1.05	0.18—5.92	0.961
**WBC Count**	<10	Ref	—	—
	>10	**1.51**	1.18—1.93	**<.001**
**Acute Renal Failure**	No	Ref	—	—
	Yes	**7.10**	1.91—26.32	**0.003**
**Platelet Count**	<150,000	Ref	—	—
	>150,000	**0.61**	0.46—0.81	**<.001**
**Return to OR**	No	Ref	—	—
	Yes	**3.82**	2.77—5.27	**<.001**

Significant variable names, associated aOR, and *p* values are in bold.

## Discussion

Traumatic brain injury is associated with high rates of morbidity and mortality. Although trauma location and its associated complications, such as bleed or fracture, along with surgical technique and approach are crucial in determining surgical adequacy, patient factors such as laboratory results and preexisting conditions provide further insight into surgical eligibility. To the best of our knowledge, this is the first study that uses NSQIP database to identify the predictors of 30-mortality and morbidity among patients undergoing traumatic brain injury procedures.

The overall mortality rate in our cohort was 20.3%. This rate is lower than two similar studies, one conducted at a university hospital in Ethiopia that evaluated all patients with TBI between 2017 and 2022 with an overall mortality of 28.8% and another study in Brazil with a mortality rate of 27.6% for patients presenting with TBI.^10,11^ This observation could be attributed to the fact that only patients who underwent surgical procedures for TBI were included in our study from the NSQIP database. Patients who are nonsurgical candidates typically have poor outcomes and higher mortality, which could explain why the mortality rate in our surgical cohort was lower than other studies. When considering studies on strictly surgical patients, a retrospective study on over 100,000 geriatric patients from the ACS National Trauma Data Bank in the United States had a mortality rate of 23.9%, which is similar to our results considering that a major proportion of our study population were elderly >60 years of age (74.48%).^11^

For the primary end-point (30-day mortality), we identified 11 preoperative variables that can effectively predict outcomes in patients undergoing neurosurgery following acute TBI. Age was one of the most significant predictors, with patients older than 80 years having the highest odds of death (up to 7.7 times) compared with the reference age group (<60 years). This is in line with studies that demonstrated that increased age is associated with increased 30-day mortality following acute TBI.^12^ Moreover, ventilator dependence, being on dialysis, having disseminated cancer, and being in a coma for more than 24 h before surgery significantly increased the risk of mortality with adjusted ORs (aORs) of 6.04, 4.97, 4.42, and 3.30, respectively. From a neurological point of view, being in a coma or requiring mechanical ventilation following trauma reflects severe brain damage with possible complications such as brain herniation due to elevated ICP, which would explain why they confer a much higher risk of mortality. In fact, several studies highlight the role of the GCS in predicting mortality after TBI.^13,14^

Although the NSQIP database does not include GCS, it does provide information about coma status, which is a surrogate for the GCS corresponding to a GCS <8. Therefore, our results suggest that a GCS score ≤8 is significantly associated with mortality. Furthermore, being on dialysis indicates severe renal impairment, which is associated with impaired platelet function and metabolic derangements which can exacerbate the damage from brain injury.^15^ Disseminated cancer induces a hypercoagulable state in the patient and weakens the immune response, which may increase mortality from a number of reasons, including thromboembolic events and infectious causes.^16,17^

Additional variables that significantly increased the odds of death were being an emergent case, having a bleeding disorder, being a diabetic, having a platelet count <150,000, having a WBC count >10,000, and having an INR > 1.2 with aORs 2.05, 1.72, 1.51, 2.04, 1.72, and 1.53, respectively. Diabetes is associated with several comorbidities that significantly increase surgical risk in general and in acute TBI in particular. Bleeding disorders, INR > 1.2, and platelets <150,000 are all factors that increase the risk of significant hemorrhage, which is very critical in patients with brain injury. It is evident from these results that a patient’s medical comorbidities (cancer, diabetes, etc.) can effectively predict the risk of mortality after surgical intervention and can be beneficial in determining surgical eligibility.

The secondary end-point, composite morbidity, was composed of four categories of outcomes in the 30-days postoperatively. Predictors of composite morbidity that were significant included hypertension, sepsis, emergency case status, WBC count, platelet count, and hematocrit. There is evident overlap in the predictors of mortality and morbidity which share similar pathophysiological mechanisms. The distinctive variables for morbidity are hypertension, sepsis, and hematocrit. Hypertension, when associated with complications such as coronary artery disease, congestive heart failure, renal failure, cerebrovascular disease, and diabetes mellitus, can increase surgical risk and morbidity.^18^

Sepsis is a life-threatening inflammatory response to bacteremia, which puts the body under significant stress and at higher risk for morbidity after surgery. A prospective cohort study, which evaluated 2.3 million patients in ACS NSQIP database at 374 U.S. hospitals between 2002 and 2012, found that preoperative sepsis increases the risk for arterial and venous thromboses, and the risk increases with severity of sepsis.^19^ The same effect on mortality is demonstrated in our results where patients who had SIRS were 1.59 (95% CI, 1.19–2.12, *p* = 0.002) times and those with sepsis were 3.27 (95% CI, 1.11–9.66, *p* = 0.032) times more likely to develop a post-op morbidity compared with those who didn’t, respectively. Finally, lower hematocrit before surgery requires transfusion of blood products, which poses the risk for hemolytic transfusion reactions and complications from large volume transfusions.^20^

The results of our study hold the highest value in situations where patient triage is of essence. It is imperative that all patients with life-threatening TBI receive surgical intervention, but in scenarios such as natural disasters and mass casualty events, where the number of patients with TBI requiring intervention overwhelms the surgical capacity of health care systems, having a robust predictive model would help triaging patients based on priority. A neurosurgeon has to decide which patient to operate on between the following two: a 75-year-old male patient with long-standing diabetes mellitus receiving hemodialysis for end-stage kidney disease and a 65-year-old male patient with no comorbidities, but whose labs reveal thrombocytopenia. Based on our model, the first patient has a significantly higher odds of death, and the second patient should be prioritized under dire circumstances.

Our study has several potential limitations. The main limitation is the lack of certain variables relevant to neurosurgery for TBI, such as the injury mechanism. The mechanism of injury remains one of the most important predictors of outcomes post brain trauma. For instance, brain injury due to high-speed motor vehicle collisions or high impact blunt trauma (such as falling from a height) confer much more damage and a higher mortality than low impact trauma, but such data were not available for analysis. Another example is GCS on presentation, which is another major predictor of outcomes. GCS is used to categorize patients into different risk groups with different prognoses. Information about the use of anticoagulants and antiplatelets was not found, which are highly relevant in trauma. Another limitation is the short follow-up period dictated by the NSQIP database (30 days). Patients with traumatic TBI often suffer long-term neurological sequelae that require long-term follow-up. The last limitation is the lack of detailed neurological findings in the database. Some patients may develop debilitating neurological symptoms such as weakness, paresthesia, aphasia, or even epilepsy, which could not be computed in our composite morbidity outcome.

## Conclusion

The predictive model at hand was established using the NSQIP database, which is a highly reliable resource for surgical data. Furthermore, the model scored high on performance testing which indicates that it can accurately predict outcomes in patients with acute TBI. The clinical value of this model lies in determining the feasibility of operating on patients with TBI and to have an accurate prognostic measure of postoperative morbidity and mortality. This is especially relevant in emergency settings such as disaster management when there is urgent need for triage and determining which patients have a better outlook in the setting of limited time and resources. This prompts decisions to be made about resource allocation and the order in which patients with TBI may be treated. Future prospective studies that include additional variables such as mechanism of injury, GCS, anticoagulant use, and neurological findings with longer follow-up intervals are needed to test this model and enhance it for better predictive capacity.

## Data Sharing Statement

Data were acquired from ACS NSQIP database for Neurosurgical procedures, which contain data queried between 2005 and 2017. For more details about data acquisition, characteristics of the analysis, and source and reproducibility of the data acquisition approaches, refer to ACS NSQIP Participant User File (PUF) 2017 using the following link: (https://www.facs.org/quality-programs/data-and-registries/acs-nsqip/participant-use-data-file/).

## Abbreviations Used

ACS NSQIP: The American College of Surgeons National Surgical Quality Improvement Program
APACHE-II score: Acute Physiology and Chronic Health Evaluation II score
AUC: area under the curve
CPT code: Current Procedural Terminology code
CVA: Cerebrovascular accident
GCS: Glasgow Coma Score
ICD-9: International Classification of Diseases Ninth Revision
ICD-10: International Classification of Diseases Tenth Revision
ICP: intracranial pressure
INR: International normalised ratio
POSSUM scale: Physiological and Operative Severity Score for the enUmeration of Mortality and Morbidity scale
ROC: Receiver operating characteristic
TBI: Traumatic brain injury
VTE: Venous thromboembolism
WBC: White blood cell count
